# Preparation and Performance Evaluation of Gelled Composite Plugging Agent Suitable for Fractured Formation

**DOI:** 10.3390/gels12010036

**Published:** 2025-12-31

**Authors:** Kecheng Liu, Kaihe Lv, Weiju Wang, Tao Ren, Jing He, Zhangkun Ren

**Affiliations:** 1 School of Petroleum Engineering, China University of Petroleum (East China), Qingdao 266580, China; lkc180716@126.com (K.L.);; 2 Oil Production Technology Research Institute of PetroChina Xinjiang Oilfield Company, Karamay 834000, China

**Keywords:** fractured formation, high-temperature and high-pressure, drilling fluid, gelled composite plugging agent, elastic gels

## Abstract

Lost circulation in fractured formations is a common yet challenging technical problem in drilling engineering. Conventional plugging methods often form sealing layers with poor stability and low pressure-bearing capacity. This study developed an efficient composite plugging agent composed of calcite particles (rigid particles), elastic gel particles, and polypropylene fibers. Utilizing a laboratory-scale fracture plugging evaluation apparatus and standard comparative experimental methods, the synergistic plugging effects of different composite systems were investigated. The results indicate that while single rigid particles can form a basic bridging structure, the pressure-bearing capacity of the resulting sealing layer is limited. Single elastic gel particles or fibrous materials struggle to effectively plug fractures of varying widths. Composite use of the plugging agents significantly enhanced the plugging performance, with the rigid/elastic/fiber ternary composite system demonstrating the best results. The optimal formulation (5% calcite particles + 3% elastic gel particles + 2% polypropylene fibers) achieved a plugging pressure-bearing capacity of 13 MPa for 2 mm-wide fractures, with a fluid loss of only 50 mL and temperature resistance up to 180 °C. Furthermore, the composite plugging agent exhibited good compatibility with the drilling fluid system and demonstrated excellent adaptability and plugging performance for fractures with different roughness levels, indicating promising potential for field application.

## 1. Introduction

Lost circulation refers to the unintended and significant loss of working fluids, such as drilling fluid or completion fluid, from the wellbore into the formation under a positive pressure differential during drilling, completion, or workover operations [1,2,3]. As a common and hazardous downhole complication in oil and gas drilling and production, lost circulation often occurs in geologically weak zones: formation interfaces, naturally fractured/vugular regions, fault zones, and low-pressure high-permeability formations [4,5,6,7]. Severe lost circulation not only causes drilling fluid loss and operational delays (thus increasing costs significantly) but also induces a rapid drop in wellbore hydrostatic pressure. This drop can lead to wellbore instability, stuck pipe, and even kicks or blowouts, posing serious risks to drilling safety [8,9,10,11,12]. Therefore, effective prevention and control of lost circulation are critical technological aspects for ensuring safe, efficient, and cost-effective drilling operations.

Studies indicate that costs associated with drilling fluid loss account for approximately 10–20% of total drilling expenditures, with 90% of these losses occurring in fractured formations [13]. Fractured formations, characterized by their complex structure, wide distribution of fracture scales, and variable connectivity, present a significant challenge in wellbore sealing operations. This difficulty is further compounded by the high-temperature and high-pressure (HTHP) conditions encountered during deep drilling [14,15]. Conventional bridging plugging agents include rigid particles such as walnut shells, flaky materials like mica flakes, and fibrous materials including cotton fibers and sawdust [16,17,18,19]. Rigid particles primarily rely on a “bridging” mechanism to form supports at fracture throats; flaky materials act by “filling” to plug micropores; and fibrous materials enhance the integrity and compactness of the plugging structure through a “network-forming” effect [20,21,22]. However, plugging agents such as walnut shells, cotton fibers, and sawdust are prone to carbonization and degradation under high-temperature conditions. This results in a significant reduction in their mechanical strength, rendering them unsuitable for plugging applications in high-temperature fractured formations. While agents such as mica flakes and shell fragments exhibit better high-temperature resistance, they often fail to effectively plug wide or complex fractures when used alone [23].

At present, plugging operations in fractured formations primarily face the following technical difficulties. On one hand, under the high-temperature conditions of deep formations, most plugging agents suffer from strength degradation. Under formation pressure, they are prone to fragmentation and instability, leading to insufficient pressure-bearing capacity of the sealing layer [24]. On the other hand, existing plugging agents exhibit poor adaptability to fractures of varying scales. The complex fracture networks with diverse morphologies make it difficult for single materials or simple composite formulations to achieve efficient plugging [25,26]. Su et al. [27] developed a novel composite plugging agent, SXM-I, using calcium carbonate, lignin fibers, and elastic materials, which demonstrated effective plugging performance in abnormally high-temperature and high-pressure carbonate reservoirs. High-temperature and high-pressure fracture plugging tests showed a drilling fluid loss of only 13.4 mL, a pressure-bearing capacity exceeding 9 MPa, and temperature resistance up to 180 °C. Yang et al. [28] developed a high-temperature resistant rigid plugging agent, HTD-2, and combined it with modified inorganic mineral fibers and elastic materials to form a composite plugging agent with a pressure-bearing capacity greater than 10 MPa and temperature resistance up to 200 °C. Bao et al. [29] developed a high-temperature resistant rigid bridging particle, SDHTP-1, and a high-temperature resistant fibrous plugging agent, SDHTF-1. By synergistically combining them with other plugging agents, they constructed a high-temperature resistant composite plugging system with a pressure-bearing capacity of 15 MPa and temperature resistance enhanced to 220 °C. Zhang et al. [30] utilized a combination of various plant fibers, rigid particles, and swelling particles to form a composite plugging agent suitable for lost circulation control while drilling. Field trials in the Liuzan area of Jidong Oilfield, China, showed that this technology significantly reduced drilling fluid loss by 15%.

Although recent studies have developed various high-temperature resistant composite plugging systems, there remain shortcomings in addressing the precise matching between particle size gradation and fracture width, as well as in understanding the synergistic mechanisms among rigid, elastic, and fibrous materials within complex fractures. This study develops an effective composite plugging agent for fractured formations. By conducting simulated fracture plugging experiments using conventional laboratory equipment, the performance of several single-component plugging agents—including rigid particles, elastic gel particles, and fibrous materials—was evaluated and optimized, with particular emphasis on the optimization of rigid particle gradation and its matching relationship with fracture width. Subsequently, the plugging performance of different composite systems was systematically studied, including rigid/fiber, rigid/elastic, elastic/fiber, and rigid/elastic/fiber combinations, to determine the optimal composite formulation. Furthermore, comprehensive evaluations of the adaptability, stability, and plugging efficiency of the composite plugging agent were carried out through drilling fluid compatibility tests and high-temperature and high-pressure full-diameter core simulation plugging experiments. The research findings provide important insights for establishing an efficient plugging technology system for fractured formations.

## 2. Results and Discussion

### 2.1. Fracture Plugging Efficiency of Single Plugging Agents

#### 2.1.1. Rigid Granular Materials

**Figure 1:**
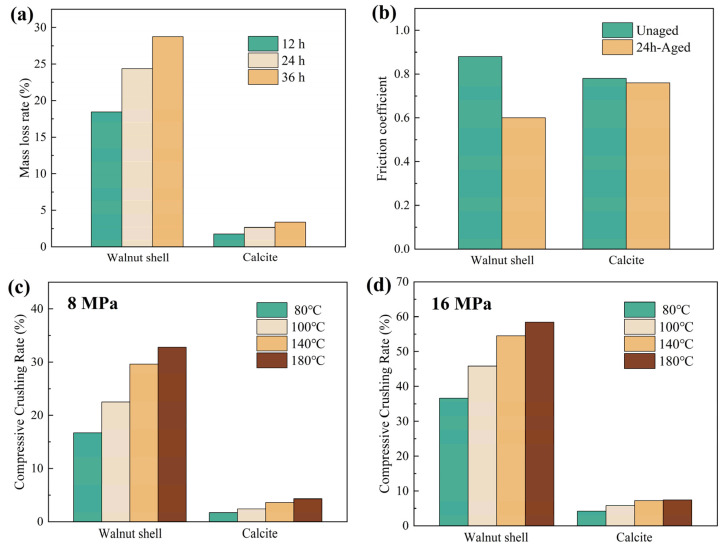
Basic performance test results of rigid granular plugging agent. (**a**) Mass loss rate of rigid particles after high-temperature aging, (**b**) the friction coefficient between rigid particles and rock samples before and after aging, the compressive crushing rate of rigid particles under (**c**) 8 MPa and (**d**) 16 MPa.

Currently, commonly used rigid granular plugging agents primarily include calcium carbonate particles and nut shell particles. Calcium carbonate particles exhibit high hardness and possess excellent temperature resistance and compressive strength. Nut shell particles, after processes such as degreasing, crushing, and screening, demonstrate strong compressive resistance and stable chemical properties. This study selected calcite particles (a type of calcium carbonate) and walnut shell particles (a type of nut shell) as representative rigid plugging agents. Their fundamental properties, such as temperature resistance and compressive strength, were tested, and the results are shown in Figure 1.

Figure 1a shows the temperature resistance of the two rigid particles. At 180 °C, the mass loss rate of walnut shell particles increased significantly with aging time: 18.46% (12 h), 24.36% (24 h), and 28.75% (36 h). In contrast, calcite particles showed much lower mass loss rates under the same conditions: 1.76%, 2.65%, and 3.37% at the corresponding time points. This indicates that the temperature resistance of calcite particles is significantly better than that of walnut shell particles. Figure 1b shows the friction coefficients between the two types of particles and the rock sample. The results indicate that after high-temperature aging, the friction coefficient of walnut shell particles decreased from 0.88 to 0.6, a reduction of 31.81%, indicating a significant decline in their retention capacity in high-temperature environments. In contrast, the friction coefficient of calcite particles changed only slightly before and after aging, decreasing from 0.78 to 0.76, a reduction of 2.56%, demonstrating better retention stability in fractures and more effective fracture plugging. Figure 1c,d show the compressive resistance of the two particles under different confining pressures. Under the same temperature and pressure conditions, calcite particles exhibited significantly lower compressive crushing rates. At 8 MPa, the crushing rate of walnut shell particles exceeded 15% across all tested temperatures; when the pressure increased to 16 MPa, their crushing rate further rose to over 35%, indicating high sensitivity to pressure. In comparison, the crushing rate of calcite particles was only 1.7–4.3% at 8 MPa and increased to only 4.2–7.4% at 16 MPa, demonstrating superior compressive strength and greater suitability for lost circulation operations in high-pressure formations.

In summary, walnut shell particles exhibit poor temperature resistance, with significant reductions in friction coefficient and compressive performance under high-temperature. Their mass loss rate reaches as high as 28.75%, indicating severe degradation under high-temperature conditions, making them unsuitable as an effective plugging material for high-temperature and high-pressure formations. In contrast, calcite particles demonstrate superior overall performance in terms of temperature resistance, retention capacity, and compressive strength. Therefore, calcite particles were selected as the rigid granular plugging agent in subsequent experiments to systematically investigate their plugging performance in different fractures and to determine the optimal particle size distribution scheme suitable for fractures of various widths. The size parameters of the plugging particles are listed in Table 1.

**Figure 2:**
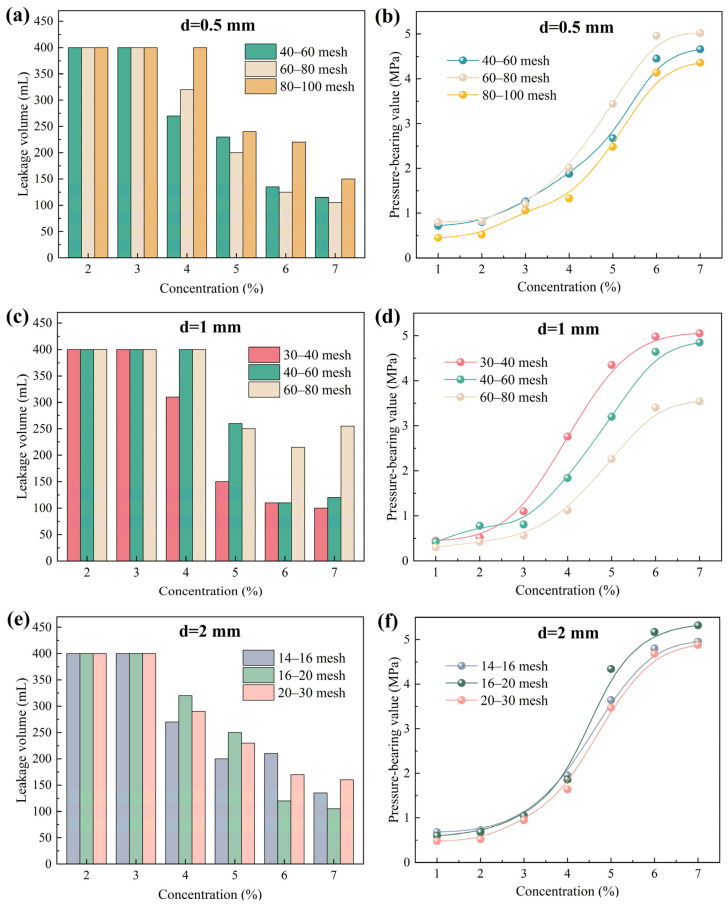
Plugging effect of calcite particles on fractures of different sizes. (**a**,**c**,**e**) show the leakage volume, while (**b**,**d**,**f**) show the pressure-bearing value.

Calcite particles of different sizes were added to 400 mL drilling fluid base slurry at specific mass-to-volume ratios. We systematically evaluated the plugging performance of calcite particles (different concentrations and mesh sizes) for 0.5 mm, 1 mm, and 2 mm fractures via high-temperature, high-pressure plugging experiments. The selection of the particle size range was primarily based on the classical “1/3 bridging rule” for fracture plugging and its derivative criteria. Three different fracture widths were chosen to simulate the typical size distribution of downhole fractures, thereby comprehensively evaluating the adaptability and effectiveness of the plugging materials under various geological conditions. The dosage of the plugging agent is based on the volume of the base slurry, and the addition is directly calculated according to the mass/volume percentage. The experimental results are shown in Figure 2. As shown in Figure 2, as the concentration of calcite particles increased, the drilling fluid loss gradually decreased, while the pressure-bearing capacity significantly improved, indicating a gradual increase in plugging success rate. This is primarily because the increased concentration of the plugging agent provides more effective particles per unit volume for bridging, making it easier to form a stable and dense sealing layer in the fractures, thereby enhancing the overall strength and integrity of the plugging structure. For the 0.5 mm fracture, 60–80 mesh calcite particles exhibited the best plugging performance, with the lowest fluid loss and the highest pressure-bearing capacity. At a particle concentration of 7%, the fluid loss was approximately 105 mL, and the pressure-bearing value reached 5.02 MPa. When the fracture width increased to 1 mm, 30–40 mesh calcite particles demonstrated superior plugging performance. At a particle concentration of 7%, the fluid loss was approximately 100 mL, and the pressure-bearing value reached 5.05 MPa. For the 2 mm fracture, 16–20 mesh calcite particles provided the best plugging effect. At a particle concentration of 7%, the drilling fluid loss was approximately 105 mL, and the pressure-bearing value reached 5.32 MPa. These results indicate that the plugging effectiveness of calcite particles depends on the matching relationship between their particle size and the fracture width. When the particle size corresponds to the fracture aperture, effective initial bridging can be achieved, leading to the formation of a dense and stable sealing layer.

**Figure 3:**
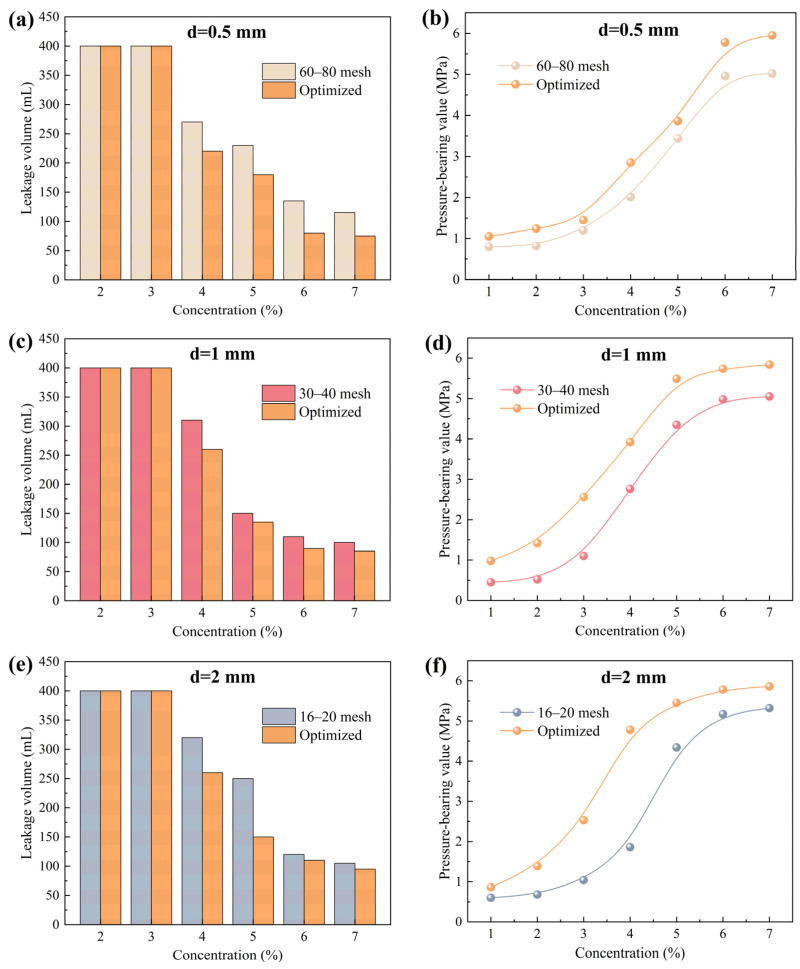
Plugging effect of calcite particle plugging agent before and after optimization on fractures of different sizes. (**a**,**c**,**e**) show the leakage volume, while (**b**,**d**,**f**) show the pressure-bearing value.

Currently, it is accepted that bridging particles of different sizes can work synergistically within fractures to form a denser and more stable sealing layer, thereby significantly enhancing the overall plugging performance [31,32,33]. To verify this view and optimize the formulation of rigid particle plugging agent, this study further conducted experiments with composite blends of calcite particles of different sizes to investigate the plugging effectiveness of the composite systems on fractures. First, linear regression analysis was performed on the “concentration-pressure-bearing value” data of single size particles, and the data points were fitted in intervals. When the goodness of fit R^2^ exceeded 0.95, it was considered that a reliable quantitative relationship between concentration and pressure-bearing value had been established. The minimum effective concentration (MEC) of the particles was then calculated using the fitting equation, which is defined as the theoretical concentration required to initiate measurable pressure-bearing plugging. The fitting results and the minimum effective plugging concentrations are listed in Table 2. Based on this, calcite particles of different sizes were blended in proportion to their MEC to formulate optimized rigid particle plugging agent compositions suitable for different fracture widths. The specific compositions are shown in Table 3. According to the optimized formulations, calcite particles were added to the drilling fluid base slurry for high-temperature and high-pressure plugging tests, and the results were compared with the plugging performance of the previously tested optimal single size systems, as shown in Figure 3. As shown in Figure 3, compared with the single size systems, the optimized formulations further reduced drilling fluid loss and improved pressure-bearing capacity. For the 0.5 mm fracture, at a 7% composite particle concentration, fluid loss decreased to 75 mL, and the pressure-bearing value increased to 5.95 MPa. For the 1 mm fracture, at the same concentration, fluid loss decreased to 85 mL, and the pressure-bearing value reached 5.84 MPa. For 2 mm fractures, fluid loss decreased to 95 mL, and the pressure-bearing value reached 5.86 MPa. The experimental results demonstrate that the graded bridging system, constructed based on the proportions of MEC, can effectively achieve a synergistic plugging mechanism of “coarse particle bridging-fine particle filling” within fractures. This leads to the formation of a sealing layer with better integrity, higher density, and stronger pressure resistance [34,35].

#### 2.1.2. Elastic Gel Particles

**Figure 4:**
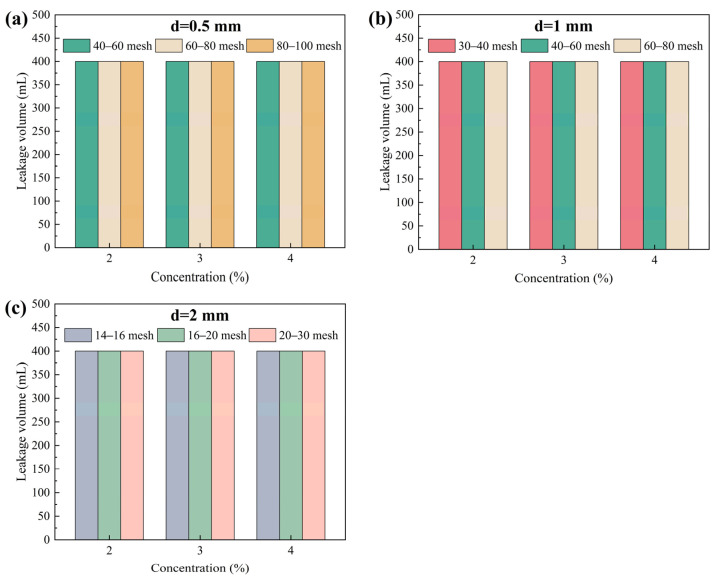
Plugging effect of elastic gel particles on fractures of different sizes. (**a**) 0.5 mm, (**b**) 1 mm, (**c**) 2 mm.

Elastic gel particles are commonly used expansive filler particles in drilling fluid lost circulation operations. They possess good deformation capability and bridging adaptability, enabling them to undergo elastic deformation under pressure and effectively fill irregular spaces within fractures, thereby enhancing the density and stability of the sealing layer [36,37]. Elastic gel particles of different mesh sizes were added to 400 mL drilling fluid base flurry at specific mass-to-volume ratios. The plugging effectiveness of particle concentration and size composition on fractures of different widths was investigated through high-temperature and high-pressure plugging experiments, and the results are shown in Figure 4. As shown in Figure 4, when the concentration of elastic gel particles of different sizes ranged from 2% to 4%, the leakage volume remained unchanged, with the entire 400 mL of test fluid being lost (100% loss rate). Effective plugging was not achieved for any of the three fracture widths, indicating that elastic gel particles alone are difficult to autonomously form a stable bridging and plugging structure. Therefore, in practical lost circulation applications, elastic gel particles need to be used in combination with other types of plugging agents to improve the overall plugging efficiency through multi-component synergy.

#### 2.1.3. Fibrous Materials

**Figure 5:**
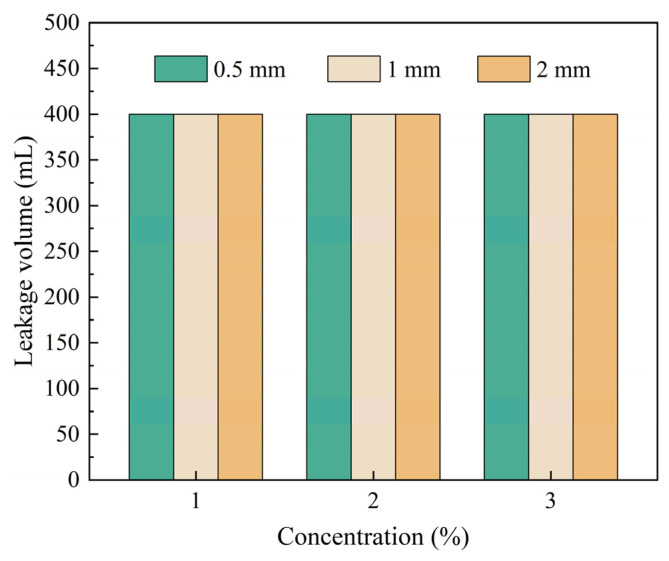
Plugging effect of polypropylene fiber on fractures of different sizes.

Fibrous materials primarily function by forming a reinforcing network during fracture plugging. They are typically used as synergistic materials with rigid bridging particles, enhancing the density and structural stability of the sealing layer through interweaving and filling, thereby achieving efficient fracture plugging [16,38]. This study selected polypropylene fiber as the fibrous plugging material due to its excellent tensile strength and temperature resistance. Its effectiveness in plugging fractures of different widths was investigated, and the results are shown in Figure 5. As shown in Figure 5, polypropylene fibers at different concentrations failed to achieve effective plugging for any of the three fracture widths. The fluid loss reached 400 mL, corresponding to a leakage rate of 100%. As expected, relying solely on polypropylene fibers cannot form a stable sealing layer within fractures. This is primarily because the fibrous material itself lacks sufficient rigid support structure and cannot autonomously establish stable mechanical bridging within the fracture. Furthermore, individual fibers struggle to be effectively retained at the fracture aperture under dynamic fluid conditions, preventing them from independently constituting a complete plugging system.

### 2.2. Fracture Plugging Efficiency of Composite Plugging Agents

From the above experimental results, it can be concluded that single type plugging agents have significant limitations in plugging fractures of different widths. Rigid particles such as calcite can achieve effective bridging through particle size matching, but their applicable range is narrow, and their plugging capacity is limited. Elastic gel particles possess good deformation and filling capabilities but struggle to independently form a stable bridging structure. Polypropylene fibers, while aiding in network formation, lack independent bridging ability. Therefore, single systems are insufficient to meet the requirements for efficient and stable fracture plugging. Subsequent experiments will focus on optimizing composite systems, investigating the plugging effectiveness of different composite plugging agents composed of rigid particles, elastic gel particles, and fibrous materials on fractures. In the experiments, the simulated fracture width was 2 mm. Calcite particles were selected in a mixed combination of 14–16 mesh, 16–20 mesh, and 20–30 mesh, with a dosage range of 3–7%. Elastic gel particles of 20–30 mesh were used at a dosage range of 2–4%. Polypropylene fibers with a length of 2.5–3 mm were applied at a dosage range of 1–3%.

#### 2.2.1. Rigid Particles and Elastic Gel Particles Combination

**Figure 6:**
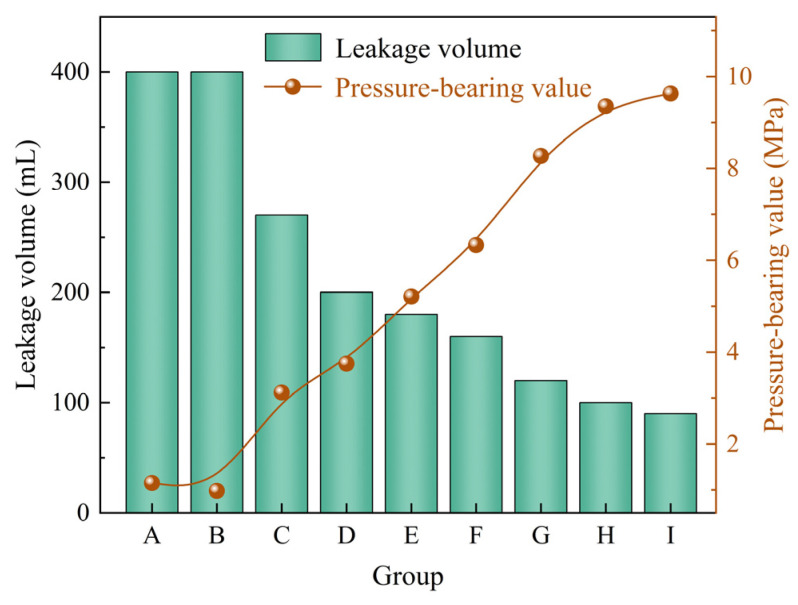
The plugging effect of rigid particles and elastic gel particles combination on 2 mm fracture.

Rigid calcite particles and elastic gel particles were combined to evaluate the plugging performance of the composite system under different concentration ratios. The experimental results are shown in Table 4. Based on the pressure-bearing values and leakage volumes presented in Table 4, Figure 6 was further plotted to more intuitively reflect the variation trends of plugging performance under different mixing ratios. From Table 4 and Figure 6, it can be observed that as the concentrations of calcite and elastic gel particles increase, the drilling fluid loss gradually decreases, while the pressure-bearing capacity of the sealing layer gradually improves. This indicates that the plugging capability of the composite system is continuously enhanced. When the calcite particle concentration is 7% and the elastic gel particle concentration is 4%, the pressure-bearing capacity of the sealing layer reaches 9.63 MPa, and the drilling fluid loss is reduced to below 100 mL, demonstrating the optimal plugging effect. Compared to single type plugging agents, the composite of rigid particles and elastic gel particles exhibits better plugging performance, which can be attributed to the effective synergy between the two types of particles within the fracture. On one hand, the rigid calcite particles first form a stable bridge in the fracture, creating a primary support framework [35]. On the other hand, the elastic gel particles deform under pressure, effectively filling the pores within the framework and adapting to the irregular surface of the fracture, thereby forming a denser and more complete sealing layer [39].

#### 2.2.2. Rigid Particles and Fibrous Materials Combination

**Figure 7:**
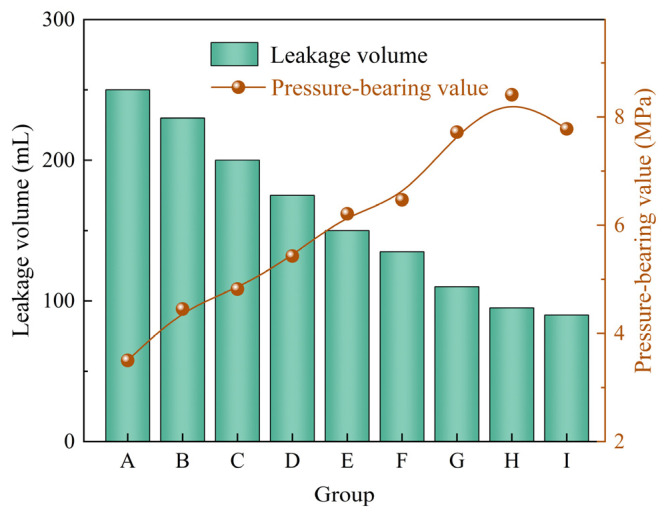
The plugging effect of rigid particles and fibrous materials combination on 2 mm fracture.

Rigid calcite particles and polypropylene fibers were combined to evaluate the plugging performance of the composite system under different concentration ratios. The experimental results are shown in Table 5. Based on the pressure-bearing values and leakage volumes presented in Table 5, Figure 7 was further plotted to more intuitively reflect the variation trends of plugging performance under different mixing ratios. From Table 5 and Figure 7, it can be observed that when the calcite concentration is ≤5%, as the concentrations of calcite and polypropylene fibers increase, the drilling fluid loss gradually decreases, and the pressure-bearing capacity of the sealing layer gradually improves. This indicates a clear synergistic enhancement effect within this range. However, when the calcite concentration increases to 7%, the pressure-bearing capacity of the sealing layer does not continue to improve with further increases in polypropylene fiber concentration, suggesting the existence of an optimal ratio range for the system. When the calcite concentration is 7% and the polypropylene fiber concentration is 2%, the pressure-bearing capacity of the sealing layer reaches 8.41 MPa, and the drilling fluid loss is reduced to below 100 mL, demonstrating the best overall plugging effect. Compared to single type plugging agents, the composite system of rigid particles and fibrous materials exhibits superior plugging performance. This is mainly attributed to the polypropylene fibers forming an interwoven network among the particles, enhancing the integrity and toughness of the sealing layer, thereby significantly improving its erosion resistance and pressure-bearing capacity [33].

#### 2.2.3. Elastic Gel Particles and Fibrous Materials Combination

**Figure 8:**
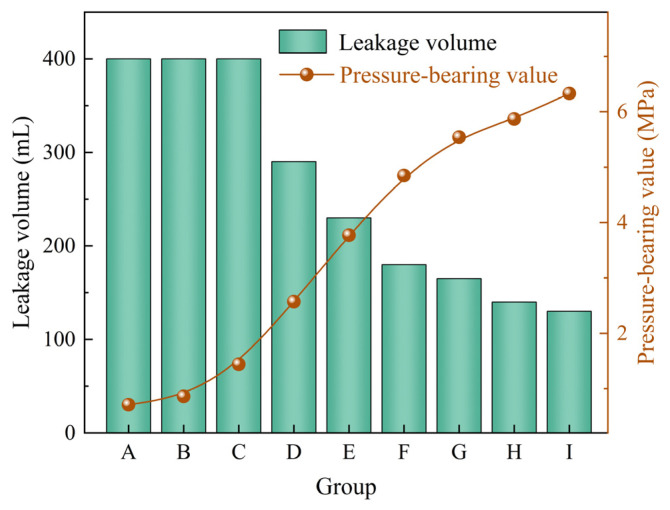
The plugging effect of elastic gel particles and fibrous materials combination on 2 mm fracture.

Elastic gel particles and polypropylene fibers were combined to evaluate the plugging performance of the composite system under different concentration ratios. The experimental results are shown in Table 6. Based on the pressure-bearing values and leakage volumes presented in Table 6, Figure 8 was further plotted to more intuitively reflect the variation trends of plugging performance under different mixing ratios. From Table 6 and Figure 8, it can be observed that when the elastic gel particle concentration is 2%, the composite system fails to form an effective sealing layer in the fracture, and the drilling fluid loss rate remains at 100%. When the elastic gel particle concentration increases to 3% and 4%, the system begins to exhibit plugging capability. As the concentrations of both materials increase, the drilling fluid loss gradually decreases, and the corresponding pressure-bearing value continuously rises, indicating improved plugging performance. When the elastic gel particle concentration is 4% and the polypropylene fiber concentration is 3%, the pressure-bearing capacity of the sealing layer reaches 6.33 MPa, and the drilling fluid loss is reduced to 130 mL, demonstrating the optimal plugging effect. Compared to single type plugging agents, the composite system of elastic gel particles and fibrous materials exhibits superior plugging performance, which is mainly attributed to the synergistic effect of the two materials. On one hand, the elastic gel particles deform under pressure, effectively filling the fracture space and achieving a tight fit. On the other hand, the polypropylene fibers form a spatial network structure through interweaving, enhancing the overall stability and impact resistance of the sealing layer, thereby constructing a sealing layer with both adaptability and toughness [39].

#### 2.2.4. Ternary Composite of Rigid/Elastic Particles and Fibrous Materials

**Figure 9:**
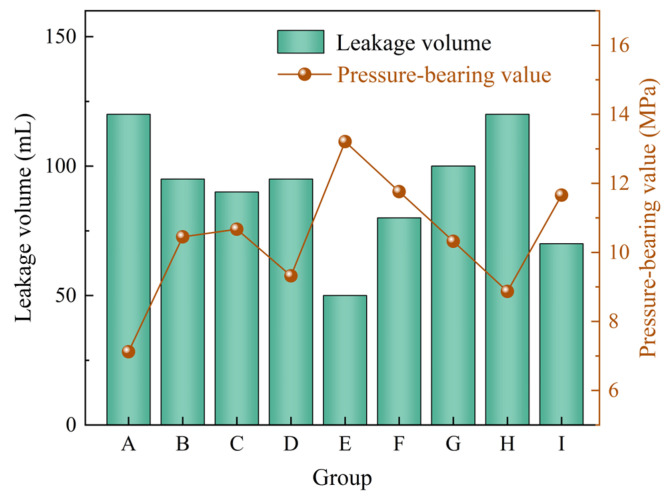
Plugging effect of rigid particles, elastic gel particles, and fibrous materials combination on 2 mm fracture.

Rigid calcite particles, elastic gel particles, and polypropylene fibers were combined to evaluate the plugging performance of the composite system under different concentration ratios. The experimental results are shown in Table 7. Based on the pressure-bearing values and leakage volumes presented in Table 7, Figure 9 was further plotted to more intuitively reflect the variation trends of plugging performance under different mixing ratios. From Table 7 and Figure 9, it can be observed that when the three materials work synergistically, the pressure-bearing capacity of the sealing layer can be significantly increased to 13.21 MPa, the fluid loss is reduced to 50 mL, and the plugging time is shortened to 5 s. This indicates a clear functional synergy and structural complementarity among the rigid particles, elastic gel particles, and fibrous materials. These results confirm that the ternary composite plugging system performs significantly better than single materials or binary composite systems. For fractures with a width of 2 mm, the optimal drilling fluid plugging formulation is determined as: 5% rigid calcite particles + 3% elastic gel particles + 2% polypropylene fibers. This combination achieved rapid, high-strength, and low-fluid-loss plugging performance in the experiments.

### 2.3. Compatibility Test with Drilling Fluid

To evaluate the compatibility of the composite plugging agent with the drilling fluid system, the rheological and filtration properties of the drilling fluid before and after adding the material were investigated. The results are shown in Table 8. From Table 8, after aging at 180 °C for 16 h, the rheological parameters (AV, PV, YP) of the drilling fluid base slurry exhibited only minor changes, and the HTHP fluid loss remained at a relatively low level (15.5 mL). This demonstrates that the base slurry itself possesses good thermal stability. After adding the composite plugging agent to the base slurry, the apparent viscosity (AV), plastic viscosity (PV), and yield point (YP) of the system increased to some extent, but the overall increase was small and did not significantly affect the fluidity of the drilling fluid. In addition, after high-temperature aging at 180 °C for 16 h, the API fluid loss and HTTP fluid loss of the drilling fluid containing the composite plugging agent were lower than those of the base slurry, indicating that the plugging agent contributes to fluid loss control under high-temperature conditions. In summary, the composite plugging agent exhibits good compatibility with the drilling fluid system. While effectively enhancing the plugging capacity, it does not negatively impact the basic properties of the drilling fluid, meeting the practical requirements for drilling operations in high-temperature, deep-fractured formations.

### 2.4. Plugging Evaluation Test on Full-Diameter Core

**Figure 10:**
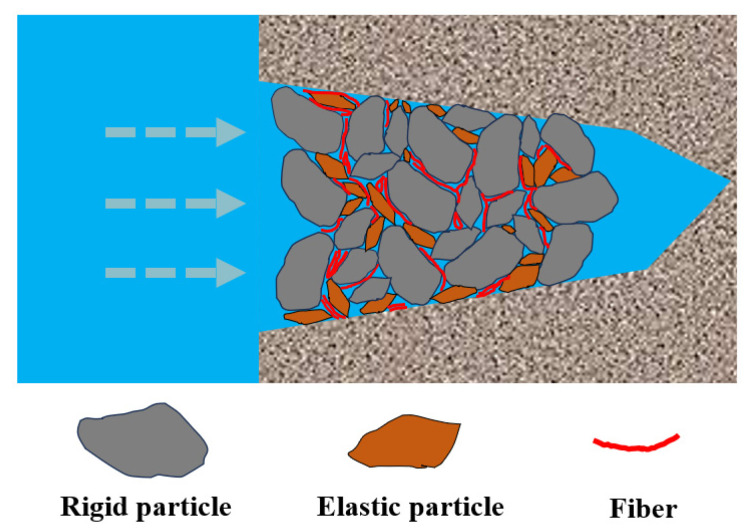
Schematic diagram of plugging mechanism of composite plugging agent.

To further verify the plugging performance and adaptability of the composite plugging agent to fractures, validation experiments were conducted using a high-temperature and high-pressure full-diameter core fracture plugging tester. The fracture width was still set at 2 mm, and three sets of fracture modules with different roughness levels were used to investigate the plugging behavior of the composite material under different fracture morphologies. Table 9 shows the fluid loss data of the plugging drilling fluid under different pressure conditions in the three modules with different roughness levels. From Table 9, it can be observed that during the gradual pressurization process in all types of fracture modules, no significant fluid loss occurred. Under a high-pressure of 13 MPa, the leakage volume remained below 30 mL, indicating that the composite plugging agent can rapidly form a dense and tough sealing layer, demonstrating good adaptability and effective high-temperature and high-pressure plugging capability for fractures with varying roughness levels. Table 10 further compares the performance of the plugging agent from this study with those reported in selected recent literature. The comparison shows that the composite plugging agent developed in this study demonstrates superior pressure-bearing capacity or temperature resistance, exhibiting outstanding overall plugging performance. From the perspective of the plugging mechanism, the composite plugging agent achieves efficient plugging in fractures primarily through the synergistic effects of the three types of materials. Rigid particles act as a structural skeleton, first bridging in the fracture to form a stable support. Elastic gel particles then deform under pressure, filling the gaps between the skeleton and enhancing interface conformity. Fibrous materials, through mutual entanglement, form a spatial network that effectively improves the structural toughness and erosion resistance of the sealing layer. These three types of materials play key roles in the three critical stages of bridging, compaction, and consolidation, collectively contributing to a significant improvement in the formation speed and overall strength of the sealing layer. The composite plugging mechanism is illustrated in Figure 10.

Overall, the composite plugging agent demonstrates favorable plugging performance, and its experimental results can have a positive impact on field operations. The agent’s ability to maintain a high-strength seal under 13 MPa pressure with a cumulative fluid loss below 50 mL indicates its potential for effectively plugging severe losses in a single application. This capability is expected to reduce non-productive time caused by repeated plugging attempts and well control incidents, thereby lowering operational costs. Furthermore, using acid-soluble calcite as the primary rigid component makes this agent suitable for production zones in carbonate or sandstone reservoirs that may require acid stimulation. The plugging zone can be removed through acidizing, eliminating the risk of permanent formation damage. Additionally, the raw materials employed (calcite, polypropylene fibers, cross-linked polymer gel, etc.) are readily available commercially, ensuring cost controllability.

However, it should be noted that this study has certain limitations. The experiments were conducted using idealized, planar fracture models. Although full-diameter core tests were incorporated to enhance geological realism, they cannot fully replicate the complexity, scale, and three-dimensional connectivity of natural fracture networks encountered in the field. The experiments were performed under constant pressure, and the long-term stability of the sealing layer under dynamic drilling conditions (e.g., fluid erosion, pressure fluctuations) was not investigated. Furthermore, the long-term coupled effects of actual downhole temperature and pressure may pose additional challenges to its performance. Therefore, future research could be further developed in the following aspects: conduct experiments using a larger-scale flow loop apparatus and more realistic fracture models to optimize the formulation of field-applicable “pills”; evaluate the long-term integrity of the sealing layer under constant temperature and pressure conditions (e.g., 7–30 days); and perform high-flow-rate erosion tests to simulate the dynamic impact of subsequent drilling fluid circulation on the formed sealing layer.

## 3. Conclusions

To address the shortcomings of existing high-temperature resistant composite plugging agents in particle-fracture matching and multi-component synergy, this study developed and evaluated a gelled composite plugging agent suitable for high-temperature fractured formations. The main research findings are as follows:

(1) Single-component systems exhibit limited plugging efficiency and poor pressure-bearing capacity. Effective plugging of wide fractures requires synergistic interaction among multiple components. The plugging performance of rigid particles is highly dependent on the match between particle size distribution and fracture width.

(2) The composite plugging agent, consisting of 5% calcite particles, 3% elastic gel particles, and 2% polypropylene fibers, achieved outstanding plugging performance through functional synergy. A stable sealing layer was constructed via the synergistic effects of rigid particle bridging, elastic gel particle deformation and filling, and fiber network reinforcement. At 180 °C, this synergy resulted in high pressure-bearing capacity (13 MPa) and low fluid loss (50 mL).

(3) The composite plugging agent demonstrated good compatibility and did not adversely affect the rheological properties of the drilling fluid. Furthermore, full-diameter core tests verified the agent’s effective adaptability to fractures with varying roughness levels, indicating strong potential for field application.

## 4. Materials and Methods

### 4.1. Materials and Instruments

Calcite particles (14–100 mesh) with a density of 2.6–2.94 g/cm^3^ and a decomposition temperature of 898.6 °C were supplied by Tianjin Ruijinte Chemicals Co., Ltd., Tianjin China. Walnut shell particles (20–50 mesh), with a compressive strength of up to 23.40 kgf, were obtained from the same supplier. Elastic gel particles (14–100 mesh), consisting of commercially available cross-linked polyacrylamide beads, were provided by Shenzhen Zhongning Technology Co., Ltd., Shenzhen, China. These gel particles have a density of 1.17–1.27 g/cm^3^, an elastic modulus of 80 MPa, and a deformation recovery rate exceeding 85%. They swell approximately 2–3 times in aqueous solution, reaching saturation within 2–4 h. Polypropylene fibers with a length of 1.5–5 mm, a diameter of 20–30 μm, and a density of 0.9–0.92 g/cm ^3^ were from Shanghai Macklin Biochemical Co., Ltd., Shanghai, China. Fracture models with slot widths of 0.5–2 mm were supplied by Qingdao Xusheng Petroleum Instrument Co., Ltd., Qingdao, China. The calcite particles were sieved, washed, and dried to remove dust and moisture. Polypropylene fibers were solvent-washed and dried to standardize their surface properties and enhance their dispersibility in water. The drilling fluid base slurry, with a density of 1.1 g/cm^3^, was provided by PetroChina Xinjiang Oilfield Company, Xinjiang, China, formulated with 3% bentonite, 0.3% Na_2_CO_3_, 0.2% NaOH, 0.1% potassium polyacrylamide (KPAM), 2% sulfomethylcellulose (SMC), 4% fluid loss additive SMP-1, and 2% high temperature stabilizer FBJ-1.

The variable frequency high-speed stirrer (GJS-1321K) and the roller heating furnace (BGRL-3) were purchased from Qingdao Tongchun Petroleum Instrument Co., Ltd., Qingdao, China. The universal testing machine (WAW-600F) was purchased from Jinan Xinshijin Testing Machine Co., Ltd., Jinan, China. The six-speed rotational viscometer (ZNN-D6A) and the high-temperature and high-pressure filter loss tester (GGS42–2A) were purchased from Qingdao Haitongda Special Instrument Co., Ltd., Qingdao, China. The high-temperature and high-pressure drilling fluid Plugging Performance Evaluation Instrument (XS-PPA) was purchased from Qingdao Xusheng Petroleum Instrument Co., Ltd., Qingdao, China. The friction coefficient meter (MCXS-2) and the high-temperature and high-pressure full-diameter core fracture plugging instrument (QYLD-1) were purchased from Haohan Well Completion & Logging Science and Technology Co., Ltd., Chengdu, China.

### 4.2. Performance Evaluation of Plugging Agent

#### 4.2.1. Temperature Resistance Test

Weigh 30 g of rigid granular plugging agent and add it to 400 mL of drilling fluid base slurry. The mixture was fully stirred for uniform dispersion, then transferred into a high-temperature aging cell and placed in a roller oven at 180 °C for 16 h, 24 h, or 36 h. After the aging cell cooled to room temperature, the plugging agent sample was removed and repeatedly rinsed with clean water to completely remove adhered drilling fluid. The cleaned plugging agent was fully dried in an oven, cooled, and weighed. The temperature resistance was evaluated by calculating the mass loss rate of the plugging agent after aging. The mass loss rate was calculated using the following formula:

$$C=\frac{M_{0}-M_{1}}{M_{0}}\times 100\%$$

In the formula, C, M_0_, M_1_ are the mass loss rate (%), the mass before aging (g), and the mass after aging (g).

#### 4.2.2. Friction Coefficient Test

A certain amount of the rigid granular plugging agent, both before and after aging, was weighed and evenly spread over the surface of a rock sample. A smaller rock sample was placed on top of the plugging agent layer, and a standard weight was added on top to apply a constant normal load. The drilling fluid base slurry was introduced at the interface between the rock sample and the plugging agent for lubrication. We horizontally pulled the upper rock sample at a constant speed using a friction coefficient meter, recorded the frictional force at the onset of relative sliding between the upper rock sample and the plugging agent, and calculated the friction coefficient. The calculation formula is as follows:

$$\mu =\frac{f}{9.8M}$$

In the formula, μ, f, M are the friction coefficient, the frictional force (N), and the total mass of the weight and the upper rock sample (g).

#### 4.2.3. Compressive Resistance Test

The rigid particle plugging agent with a particle size of 20–30 mesh was soaked in clear water, and then placed in a roller heating furnace, and hot rolled for 16 h at different temperatures. After the plugging agent was taken out, uniaxial compressive tests were performed under specified confining pressures of 8 MPa and 16 MPa, respectively, and the pressure was maintained for 5 min. After the pressure is released, the plugging agent is transferred to a 50-mesh sieve for screening to separate the uncrushed particles. According to the mass of the residue on the screen, the crushing rate of the plugging agent is calculated. The calculation formula is as follows:

$$K=\frac{M_{1}-M_{2}}{M_{1}}\times 100\%$$

In the formula, K, M_1_, M_2_, are the compressive crushing rate (%), the mass of plugging agent before pressure (g), and the mass of remaining plugging agent after pressure.

### 4.3. Fracture Plugging Experiment

**Figure 11:**
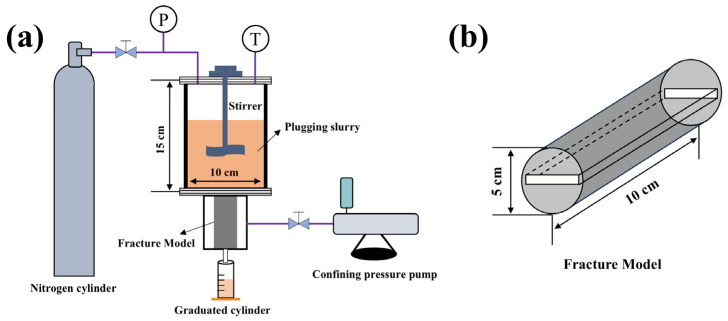
(**a**) High-temperature and high-pressure drilling fluid plugging evaluation device structure diagram and (**b**) structure of the fracture model.

Firstly, a certain amount of plugging agent was added to 400 mL drilling fluid base slurry, and the plugging slurry was prepared after stirring evenly. Then, the plugging performance evaluation device of high-temperature and high-pressure drilling fluid (Figure 11) was used to evaluate the pressure-bearing capacity of the plugging agent to simulated fractures at 180 °C. In the experiment, different specifications (0.5–2 mm) of fracture plates were selected to simulate formation fractures with different widths. The experimental steps are as follows: (1) The selected specifications of the slit plate are loaded into the gripper and installed on the high-temperature and high-pressure reaction kettle. (2) Pour the prepared plugging slurry into the kettle, apply a confining pressure of 3.5 MPa, open the heating system, and adjust the speed of the stirrer to simulate the dynamic plugging process. (3) Nitrogen was introduced and slowly pressurized to 0.5 MPa, and the step pressure test was started from 0.5 MPa. (4) Stand at each pressure point for 10 min to observe whether there is drilling fluid outflow at the fracture plate outlet. If there is no fluid outflow at the outlet of the fracture plate, indicating that the plugging is successful, then continue to slowly pressure to the next pressure point (increasing by 0.5–1 MPa), and repeat the standing and observation steps, and continue to pressure until the sealing layer breaks through. The testing procedure was designed with reference to the standard methods for evaluating lost circulation materials as per API Recommended Practice 13B.

The criterion for judging the breakthrough of the sealing layer is as follows: under a certain pressure, if the leakage velocity remains stable within 10 s and is greater than 50 mL/s, it is regarded as the failure of the sealing layer. At this time, the previous experimental pressure point is the pressure-bearing capacity value of the sealing layer.

### 4.4. Compatibility Test with Drilling Fluid

The optimized plugging agent formula is added to the drilling fluid base slurry, and the drilling fluid system is prepared after mixing evenly. The rheological properties and filtration properties of the drilling fluid were measured by viscometer and high-temperature and high-pressure filter loss tester, respectively, and compared with the drilling fluid without plugging agent to investigate the compatibility of plugging agent and drilling fluid. Among them, the test condition of API filtration loss is 25 °C × 0.7 MPa, and the test condition of HTHP filtration loss is 180 °C × 3.5 MPa.

### 4.5. Plugging Evaluation Test on Full-Diameter Core

The plugging evaluation experiment was carried out by using the high-temperature and high-pressure full-diameter core fracture plugging instrument. The steps are as follows: (1) The optimized plugging agent formula was added to the drilling fluid base slurry, and the plugging drilling fluid was prepared after mixing evenly. (2) The full-diameter core was loaded, and the confining pressure was increased to 3.5 MPa. (3) Inject plugging drilling fluid, open the stirring device, connect the pipeline. (4) The flow pressure is set to 3.5 MPa, and after 30 s of stability, the switch at the outlet is opened. (5) When the leakage is 0 mL, the current pressure is maintained for 10 min. If leakage occurs again within 10 min, the leakage time and leakage amount are recorded. When the leakage amount is 0 mL again, the voltage is stabilized for 10 min. (6) After 10 min of pressure stabilization, the pressure was 1–2 MPa, and step 5 was repeated until the plugging failed and the experiment was completed.

## Figures and Tables

**Table 1:** The size parameters of the plugging particles.

Size (mesh)	14–16	16–20	20–30	30–40	40–60	60–80	80–100
Diameter (mm)	1.4–1.18	1.18–0.9	0.9–0.6	0.6–0.45	0.45–0.3	0.3–0.2	0.2–0.15

**Table 2:** Fitting parameters and MEC of single size calcite particles for different fracture widths.

Fracture Size (mm)	Particle Size (Mesh)	Pressure-Bearing Value (MPa)							Fitting Equation	R^2^	MEC (%)
		1%	2%	3%	4%	5%	6%	7%			
0.5	40–60	0.72	0.80	1.26	1.88	2.67	4.45	4.66	y = 1.29x − 3.42	0.953	2.67
	60–80	0.80	0.82	1.20	2.01	3.44	4.96	5.02	y = 1.27x − 2.82	0.983	2.22
	80–100	0.45	0.52	1.06	1.33	2.48	4.14	4.36	y = 1.41x − 4.38	0.989	3.11
1.0	30–40	0.45	0.52	1.10	2.76	4.35	4.98	5.05	y = 1.32x − 2.66	0.966	2.01
	40–60	0.42	0.78	0.81	1.84	3.20	4.64	4.85	y = 1.09x − 2.37	0.965	2.18
	60–80	0.30	0.42	0.56	1.12	2.26	3.40	3.54	y = 0.97x − 2.51	0.979	2.60
2.0	14–16	0.68	0.72	1.05	1.95	3.64	4.80	4.95	y = 1.08x − 1.87	0.952	1.74
	16–20	0.60	0.68	1.04	1.86	4.34	5.17	5.32	y = 1.49x − 3.59	0.953	2.41
	20–30	0.48	0.52	0.95	1.64	3.47	4.68	4.88	y = 1.30x − 3.17	0.974	2.44

Note: The linear fitting follows y = a · x + b, where y is the pressure-bearing value (MPa) and x is the particle concentration (%).

**Table 3:** Optimized formulations of rigid particle plugging agent for different fracture sizes.

Fracture Size (mm)	Optimized Formulation
0.5	33% (40–60 mesh) + 28% (60–80 mesh) + 39% (80–100 mesh)
1.0	30% (30–40 mesh) + 32% (40–60 mesh) + 38% (60–80 mesh)
2.0	26% (14–16 mesh) + 37% (16–20 mesh) + 37% (20–30 mesh)

**Table 4:** Rigid particles and elastic gel particles combination plugging 2 mm fracture experimental results.

Test No.	Calcite Particle Concentration (%)	Elastic Gel Particle Concentration (%)	Pressure-Bearing Value (MPa)	Plugging Time (s)	Leakage Volume (mL)
A	3.0	2.0	1.15	N/A	400
B	3.0	3.0	0.98	N/A	400
C	3.0	4.0	3.12	82	275
D	5.0	2.0	3.75	62	200
E	5.0	3.0	5.21	76	185
F	5.0	4.0	6.33	45	160
G	7.0	2.0	8.27	53	120
H	7.0	3.0	9.35	24	105
I	7.0	4.0	9.63	20	95

**Table 5:** Rigid particles and fibrous materials combination plugging 2 mm fracture experimental results.

Test No.	Calcite Particle Concentration (%)	Polypropylene Fiber Concentration (%)	Pressure-Bearing Value (MPa)	Plugging Time (s)	Leakage Volume (mL)
A	3.0	1.0	3.50	75	250
B	3.0	2.0	4.45	32	230
C	3.0	3.0	4.82	28	200
D	5.0	1.0	5.43	40	175
E	5.0	2.0	6.21	21	150
F	5.0	3.0	6.47	23	135
G	7.0	1.0	7.72	35	110
H	7.0	2.0	8.41	20	95
I	7.0	3.0	7.78	18	90

**Table 6:** Elastic gel particles and fibrous materials combination plugging 2 mm fracture experimental results.

Test No.	Elastic Gel Particle Concentration (%)	Polypropylene Fiber Concentration (%)	Pressure-Bearing Value (MPa)	Plugging Time (s)	Leakage Volume (mL)
A	2.0	1.0	0.71	N/A	400
B	2.0	2.0	0.86	N/A	400
C	2.0	3.0	1.44	N/A	400
D	3.0	1.0	2.57	95	290
E	3.0	2.0	3.77	84	230
F	3.0	3.0	4.85	77	180
G	4.0	1.0	5.54	80	165
H	4.0	2.0	5.87	68	140
I	4.0	3.0	6.33	55	130

**Table 7:** Rigid particles, elastic gel particles, and fibrous materials combination plugging 2 mm fracture experimental results.

Test No.	Calcite Particle Concentration (%)	Elastic Gel Particle Concentration (%)	Polypropylene Fiber Concentration (%)	Pressure-Bearing Value (MPa)	Plugging Time (s)	Leakage Volume (mL)
A	3.0	2.0	1.0	7.12	27	120
B	3.0	3.0	2.0	10.45	20	95
C	3.0	4.0	3.0	10.67	18	90
D	5.0	2.0	3.0	9.32	15	95
E	5.0	3.0	2.0	13.21	5	50
F	5.0	4.0	1.0	11.76	10	80
G	7.0	2.0	3.0	10.32	14	100
H	7.0	3.0	1.0	8.87	18	120
I	7.0	4.0	2.0	11.66	12	70

**Table 8:** Effect of composite plugging agent on the basic properties of drilling fluid.

Drilling Fluid System	Experimental Condition	AV/(mPa·s)	PV/(mPa·s)	YP/(Pa)	FL(API)/mL	FL(HTHP)/mL
base slurry	unaged	68	61	12	N/A	N/A
	16 h-aged	61	55	9	3.8	15.5
with plugging agent	unaged	75	65	14	N/A	N/A
	16 h-aged	67	59	11	3.2	13.3

**Table 9:** Experimental results of full-diameter core plugging.

Test No.	Drilling Fluid Formula	Leakage Volume (mL)					
		5 MPa	7 MPa	9 MPa	10 MPa	12 MPa	13 MPa
1	base slurry + 5% rigid calcite particles + 3% elastic gel particles + 2% polypropylene fiber	17	20	14	20	26	24
2		0	0	5	0	12	15
3		0	0	0	5	0	10

**Table 10:** Performance comparison of composite plugging agents in fractures.

No.	Plugging Agent Formulation	Fracture Size (mm)	Pressure-Bearing Value (MPa)	Temperature Resistance (°C)	Ref.
1	calcite particles + elastic gel particles + polypropylene fibers	2	13	180	This work
2	rigid material + lignin fiber + elastic material + calcium carbonate	2	9	180	Su et al. [27]
3	rigid material + fiber + elastic material	2	10	200	Yang et al. [28]
4	inorganic plugging material + fiber + elastic particles + rigid particles	not mentioned	8	130	Zhang et al. [30]

## Data Availability

The original contributions presented in this study are included in the article. Further inquiries can be directed to the corresponding author.

## References

[1] Jaf P.T., Razzaq A.A., Ali J.A. (2023). The state-of-the-art review on the lost circulation phenomenon, its mechanisms, and the application of nano and natural LCM in the water-based drilling fluid. Arab. J. Geosci..

[2] Yang J., Sun J., Bai Y., Lv K., Zhang G., Li Y. (2022). Status and prospect of drilling fluid loss and lost circulation control technology in fractured formation. Gels.

[3] Abrams A. (1977). Mud design to minimize rock impairment due to particle invasion. J. Pet. Technol..

[4] Sun J., Bai Y., Cheng R., Lyu K., Liu F., Feng J., Lei S., Zhang J., Hao H. (2021). Research progress and prospect of plugging technologies for fractured formation with severe lost circulation. Pet. Explor. Dev..

[5] Elkatatny S., Ahmed A., Abughaban M., Patil S. (2020). Deep illustration for loss of circulation while drilling. Arab. J. Sci. Eng..

[6] Sun J., Yang J., Lv K., Bai Y., Liu J., Huang X. (2025). Research Status and Prospect of Deep and Ultra-deep Drilling Technology. Xin-Jiang Oil Gas.

[7] Kulkarni S.D., Jamison D.E., Teke K.D., Savari S. (2016). Managing suspension characteristics of lost-circulation materials in a drilling fluid. SPE Drill. Complet..

[8] Winn C., Dobson P., Ulrich C., Kneafsey T., Lowry T.S., Akerley J., Delwiche B., Samuel A., Bauer S. (2023). Context and mitigation of lost circulation during geothermal drilling in diverse geologic settings. Geothermics.

[9] Yang H., Li H., Xu H., Wang R., Zhang Y., Xing L., Chen X., Peng L., Kang W., Sarsenbekuly B. (2026). Enhanced CO_2_ foam stabilization with fluorescent nano polymer microspheres for improved oil recovery: Insights from microscopic and macroscopic displacement studies. Geoenergy Sci. Eng..

[10] Masi S., Molaschi C., Zausa F., Michelez J. (2011). Managing circulation losses in a harsh drilling environment: Conventional solution vs. CHCD through a risk assessment. SPE Drill. Complet..

[11] Huang X., Zhang X., Yuan Z., Zhang Y. (2024). Research Status of Wellbore Stabilization Drilling Fluid Materials in Complex Shale Formations. Xin-Jiang Oil Gas.

[12] Idress M., Hasan M.L. (2020). Investigation of different environmental-friendly waste materials as lost circulation additive in drilling fluids. J. Pet. Explor. Prod. Technol..

[13] Vivas C., Salehi S. (2021). Rheological investigation of effect of high temperature on geothermal drilling fluids additives and lost circulation materials. Geothermics.

[14] Aston M.S., Alberty M.W., McLean M.R., De Jong H.J., Armagost K. Drilling fluids for wellbore strengthening. Proceedings of the SPE/IADC Drilling Conference and Exhibition.

[15] Amanullah M. Characteristics, Behavior and performance of ARC plug date Seed-based sized particulate LCM. Proceedings of the SPE Kingdom of Saudi Arabia Annual Technical Symposium and Exhibition.

[16] Liu F., Liu Y., Zhang Z., Li Y., Liu C., Ma Z. (2024). A Machine Learning-Based Bridging Particle Size Recommendation Method for Lost Circulation Control. Xin-Jiang Oil Gas.

[17] Alsaba M., Nygaard R., Saasen A., Nes O.M. (2016). Experimental investigation of fracture width limitations of granular lost circulation treatments. J. Pet. Explor. Prod. Technol..

[18] Shuttleworth N., Frederiks K., DerPlas K.V., McCraith S., Taoutaou S. An innovative inert material to cure the losses in the brent depleted reservoirs—North Sea Case Histories. Proceedings of the Abu Dhabi International Petroleum Exhibition and Conference.

[19] Zhang G., Yang S., Li P. (2024). Synthesis and Evaluation of Modified Sulfomethylated Phenolic Resin MSP-1. Drill. Fluid Complet. Fluid.

[20] Moradi-Araghi A. (2000). A review of thermally stable gels for fluid diversion in petroleum production. J. Pet. Sci. Eng..

[21] Bruton J.R., Ivan C.D., Heinz T.J. Lost circulation control: Evolving techniques and strategies to reduce downhole mud losses. Proceedings of the SPE/IADC Drilling Conference and Exhibition.

[22] Mirabbasi S.M., Ameri M.J., Alsaba M., Karami M., Zargarbashi A. (2022). The Evolution of Lost Circulation Prevention and Mitigation Based on Wellbore Strengthening Theory: A Review on Experimental Issues. J. Pet. Sci. Eng..

[23] Sengupta B., Sharma V.P., Udayabhanu G. (2012). Gelation studies of an organically cross-linked polyacrylamide water shut-off gel system at different temperatures and pH. J. Pet. Sci. Eng..

[24] Soomro N.A., Ansari U., Shams B., Memon M.K., Bhutto D.K., Rui Z., Pan Y. (2025). Experimental assessment of the stability and impact of water-based fracturing fluid with and without Triethanolamine (TEA). Fuel Commun..

[25] García-Uriostegui L., Pineda-Torres G., López-Ramírez S., Barragán-Aroche J., Durán-Valencia C. (2017). Inverse emulsion free-radical polymerization of acrylamide terpolymer for enhanced oil recovery application in harsh reservoir conditions. Polym. Eng. Sci..

[26] Verret R., Robinson B., Cowan J., Fader P., Looney M. Use of micronized cellulose fibers to protect producing formations. Proceedings of the SPE International Conference and Exhibition on Formation Damage Control.

[27] Su X., Lian Z., Fang J., Xiong H., Wu R., Yuan Y. (2019). Lost circulation material for abnormally high temperature and pressure fractured-vuggy carbonate reservoirs in Tazhong block, Tarim Basin, NW China. Pet. Explor. Dev..

[28] Yang Y., Zhang J., Zhang L., Shang Y., Wang L., Zhang H. (2025). A Composite Plugging Materials for Drilling in High-temperature Deep Fractured Formations. Oilfield Chem..

[29] Bao D., Qiu Z., Qiu W., Wang B., Guo B., Wang X., Liu J., Chen J. (2019). Experiment on properties of lost circulation materials in high temperature formation. Acta Pet. Sin..

[30] Zhang Y.X., Wang Z.M., Xu J., Shen Y.Y., Zheng Z., Fan X.J. (2021). Development and Evaluation of a Composite Drilling Plugging Agent. International Field Exploration and Development Conference.

[31] Kong Y. (2022). Study and Application of Formation Environment Responsive Plugging Material. Drill. Fluid Complet. Fluid.

[32] Zhu D., Xu Z., Sun R., Fang X., Gao D., Jia X., Hu J., Weng J. (2021). Laboratory evaluation on temporary plugging performance of degradable preformed particle gels (DPPGs). Fuel.

[33] Zhai K., Yi H., Liu Y., Geng Y., Fan S., Zhu D. (2020). Experimental Evaluation of the Shielded Temporary Plugging System Composed of Calcium Carbonate and Acid-Soluble Preformed Particle Gels (ASPPG) for Petroleum Drilling. Energy Fuels.

[34] Fazelabdolabadi B., Khodadadi A.A., Sedaghatzadeh M. (2015). Thermal and rheological properties improvement of drilling fluids using functionalized carbon nanotubes. Appl. Nanosci..

[35] You L., Zou J., Kang Y., Bai R., Liu Y., Tan W., Li X. (2024). Mechanisms of Formation Damage by Lost Drilling Fluids in Fractured Tight Metamorphic Rock Gas Reservoirs. Drill. Fluid Complet. Fluid.

[36] Xu C., Kang Y., You L., You Z. (2017). Lost-circulation control for formation-damage prevention in naturally fractured reservoir: Mathematical model and experimental study. SPE J..

[37] Qiao M., Zhu Z., Yan K. (2025). Research and Application Status of Plugging Materials for Drilling Fluid. Xin-Jiang Oil Gas.

[38] Ramasamy J., Amanullah M. Novel fibrous lost circulation materials derived from deceased date tree waste. Proceedings of the SPE Kingdom of Saudi Arabia Annual Technical Symposium and Exhibition.

[39] Ren J., Xu P., Xu M., Zhang Y., Wang X. (2022). Research on the Development and Plugging Capacity of a Honeycomb Porous Lost-Circulation Material. ACS Omega.

